# Reducing infections through commissioning

**DOI:** 10.1186/1753-6561-5-S6-P325

**Published:** 2011-06-29

**Authors:** H Loveday, J Steiner

**Affiliations:** 1 Richard Wells Research Centre, University of West, Brentford, UK

## Introduction / objectives

The DH (England) Cleaner Hospitals Programme (CHP) had a major impact on reducing cases of MRSA bacteraemia and *C. diff.* infections in NHS acute hospitals. Mandatory surveillance data and CHP feedback indicate that pre-48 and 72 hour MRSA and *C. diff* cases reduced at a slower pace. Commissioning organisations (CO) were key in driving acute care reductions in HAI through contract indicators but the nature of primary and community care (PCC) made it difficult to achieve similar outcomes. This study explored: innovation in commissioning to reduce HAI across the healthcare economy (HCE); challenges in reducing priority organisms in PCC; strategies to engage non-acute providers to bring about reductions in HAI.

## Methods

A case study method was used. The CHP team provided data of four CO with difficulty in reducing priority organisms and four that appeared to be having greater success. A purposive sample was selected to provide insights from rural, urban and metropolitan CO. Data were collected using individual and focus group interviews with key informants and reviews of documentation. Data were analysed using a framework process.

## Results

Barriers to engaging PCC practitioners in initiatives to reduce HAI include: limited levers to drive PCC improvement activity; practitioner contracts; weak evidence that priority organisms are acquired in non-acute care; lack of shared learning. Mature cross-HCE groups seeking system wide solutions such as antimicrobial prescribing and elective screening for MRSA were more successful. Timely use of national and local surveillance and audit data to alert CO to potential problems and guide intervention was key.

## Conclusion

Commissioning for HAI reduction across the HCE is challenging. There is evidence that separating CO from provider activity might be of benefit. There is less infection prevention resource and activity within PCC to drive improvement.

## Disclosure of interest

H. Loveday Grant/Research support from the Department of Health Policy Research Programme, J. Steiner Grant/Research support from the Department of Health Policy Research Programme.

